# Supplementary material for: Controlled noninvasive modulation of deep brain regions in humans
Source: Commun Eng. 2024 Jan 12;3:13. doi: 10.1038/s44172-023-00146-4 (PMC10956068; doi:10.1038/s44172-023-00146-4)
Supplement: Supplementary file 2 — Supplementary Information [file 44172_2023_146_MOESM2_ESM.pdf]

Supplementary Material

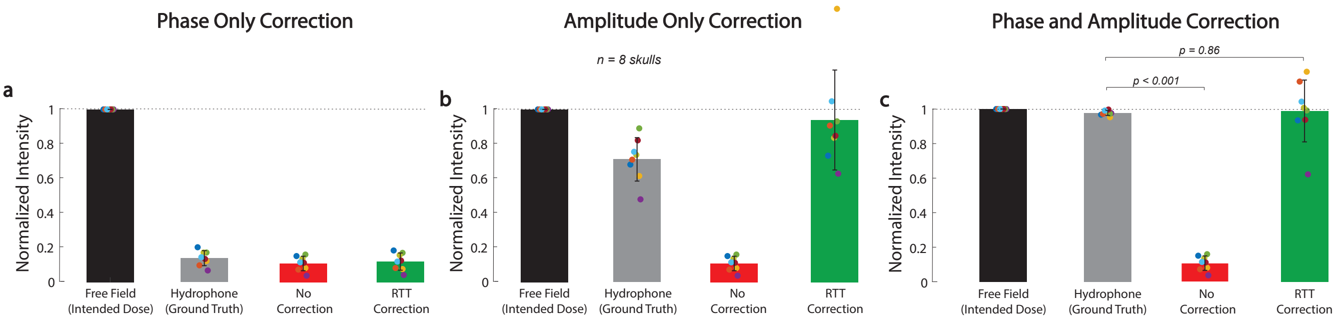

Supplementary Figure 1. Correction for phase is insufficient to account for the skull attenuation. Spatial peak intensity at the central target using the phase correction component of RTT only (A), the amplitude component only (b) and both (c). Same format as in Fig. 3.

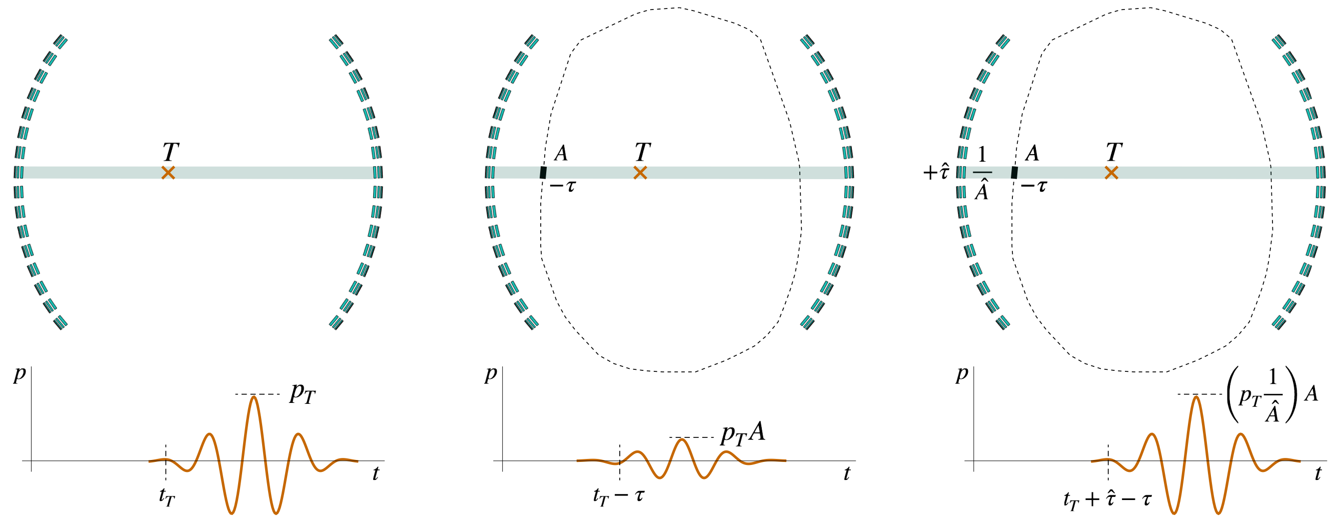

Supplementary Figure 2. The correction concept illustrated. The correction uses through-transmit measurements to measure the attenuation A and speedup τ associated with each segment of the skull within the beam connecting each transmitting and receiving elements. These measurements are performed through all possible pairs. The resulting matrix can be "inverted" (see the algorithm description for specifics) to provide A_i and τ_i values for each element. These values are then used to scale and delay the emission of the ultrasound from the respective element.

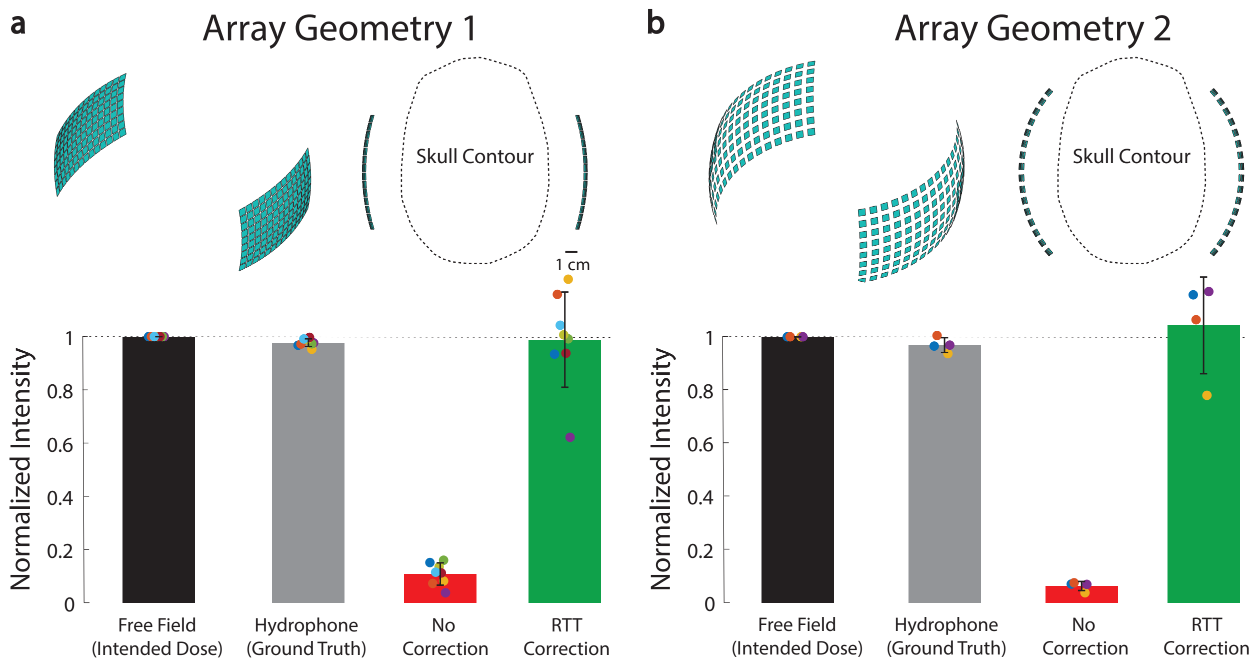

Supplementary Figure 3. RTT is robust across distinct hardware. (**A**) Array geometry used in the main text. The associated correction values are the same data as in Fig. 3. (**b**) Array geometry with a larger aperture and the associated correction data. See Methods for the specific dimensions.

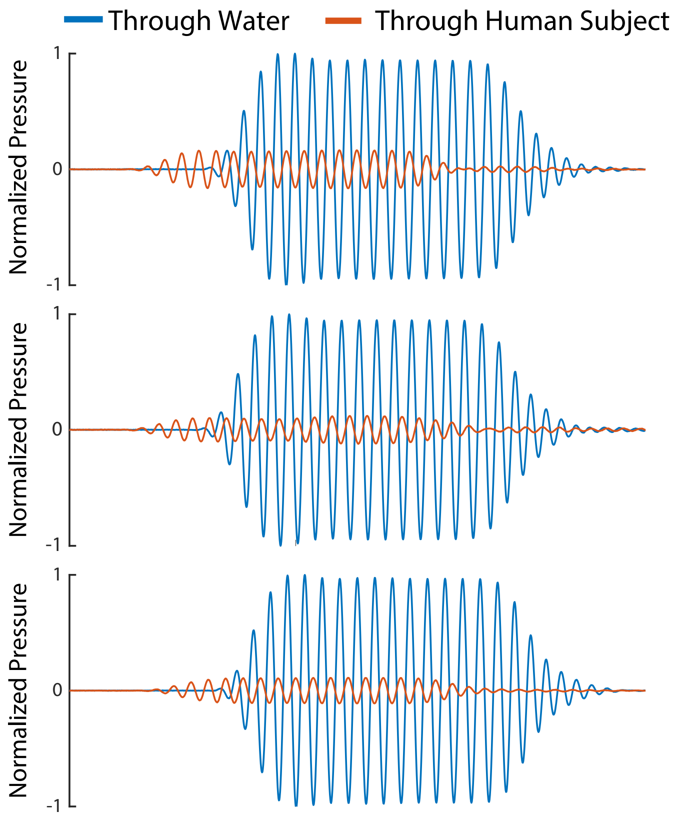

Supplementary Figure 4. Example through-transmit signals recorded in a human subject. Received waveforms on three separate elements in water (blue) and after passing through the head (orange).

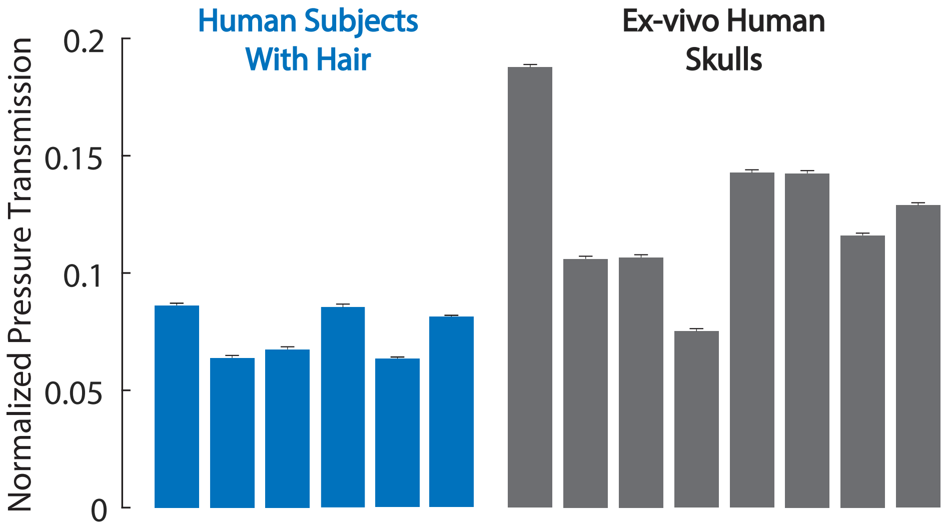

Supplementary Figure 5. RTT applied through the human head.
Average through-transmit attenuation value across all elements in 6 human subjects and 8 *ex-vivo* human skulls. No hair shaving was necessary to obtain robust through-transmit signals (see also Suppl. Fig. 4).

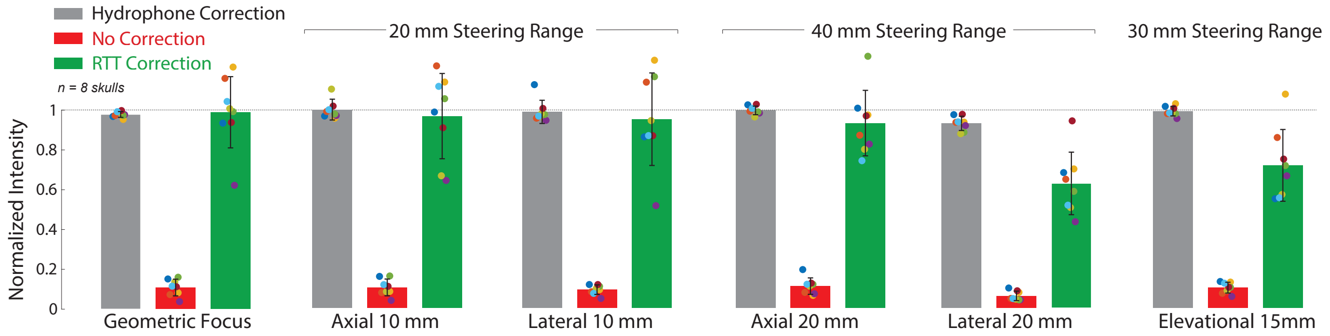

Supplementary Figure 6. RTT performance as a function of the range of action.
Same format as in Fig. 3, now also including targets comprising the full steering range of the array: 10 mm axial, 20 mm axial, 10 mm lateral, 20 mm lateral, and 15 mm elevational with respect to the central target. The axial direction refers to the line connecting the centers of the two transducers. Additional dynamic range can be provided by physically translating the arrays.

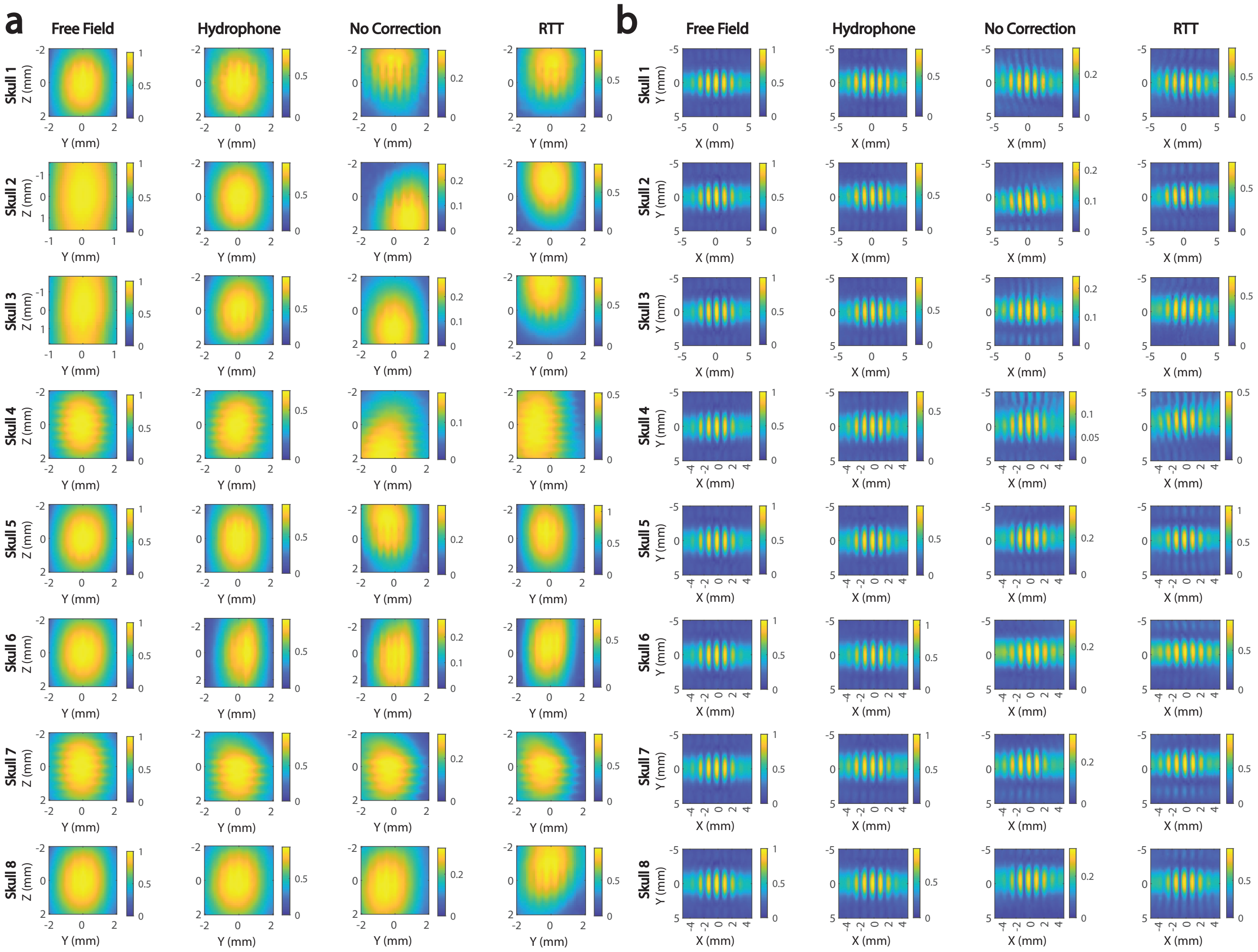

Supplementary Figure 7. Ultrasound pressure fields at central target for all correction cases, skulls, and field dimensions. Same format as in Fig. 3.

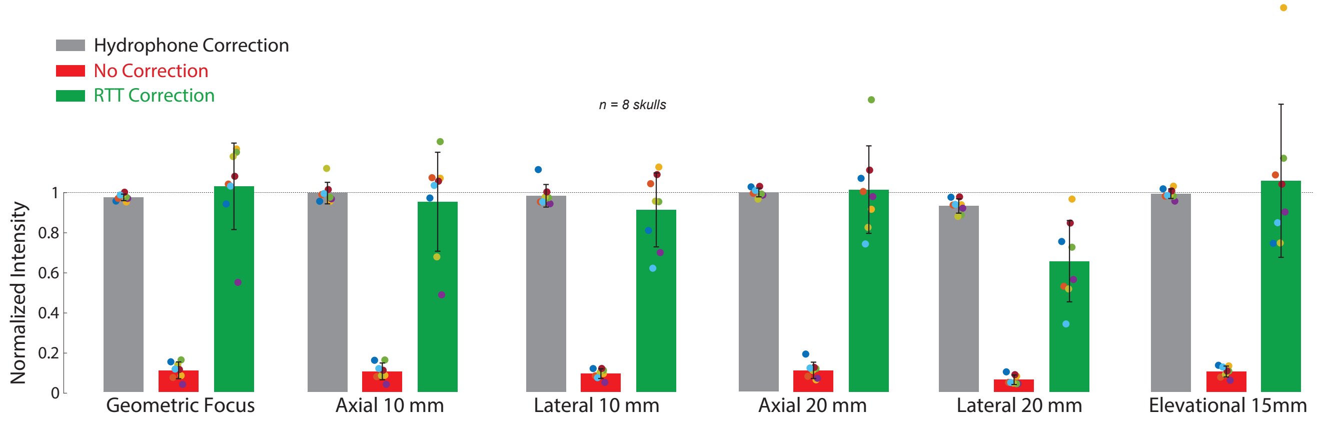

Supplementary Figure 8. RTT correction using all through-transmit pairs.
Correction results for no target-dependent weighting (see Methods). In this case, the correction is target-independent. The correction is calculated once, and then applied to any target location.

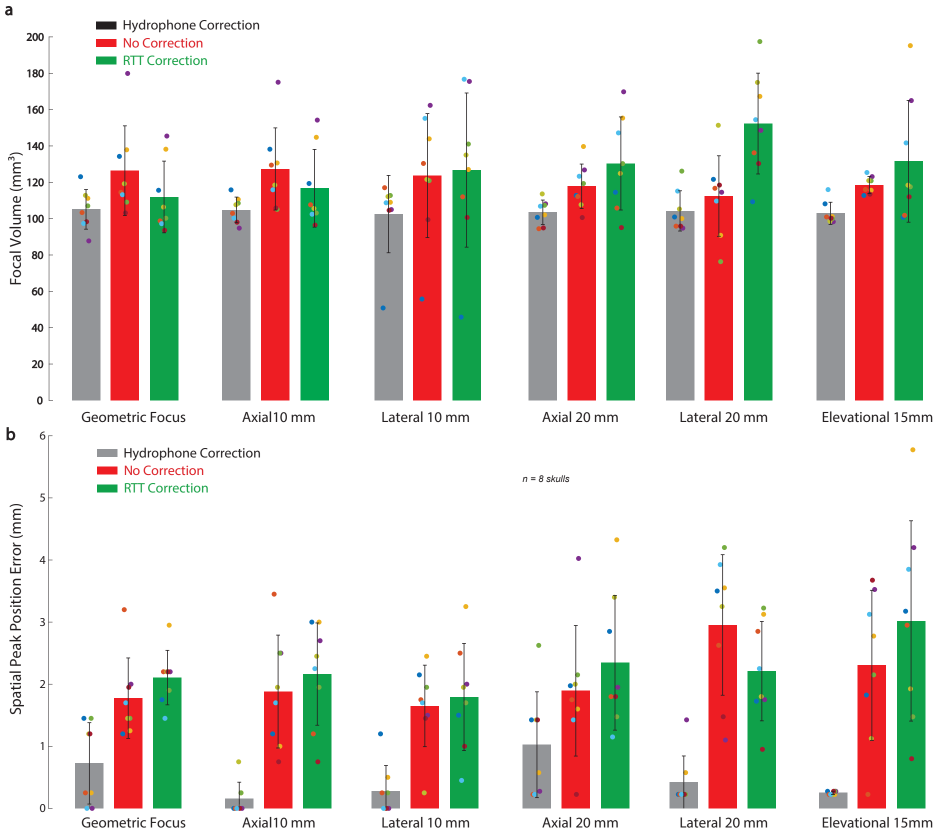

Supplementary Figure 9. Field volume and target localization for each correction.

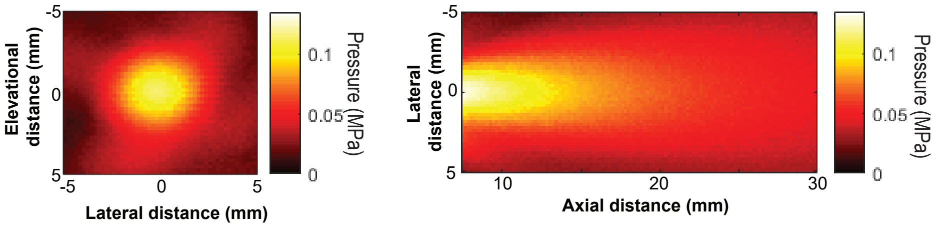

Supplementary Figure 10. Pressure field produced by each individual element.
Pressure field of single element of the transducer phased arrays measured with hydrophone field scan. Elevational-Lateral plane was taken at an axial distance of 7.5 mm from the transducer face.

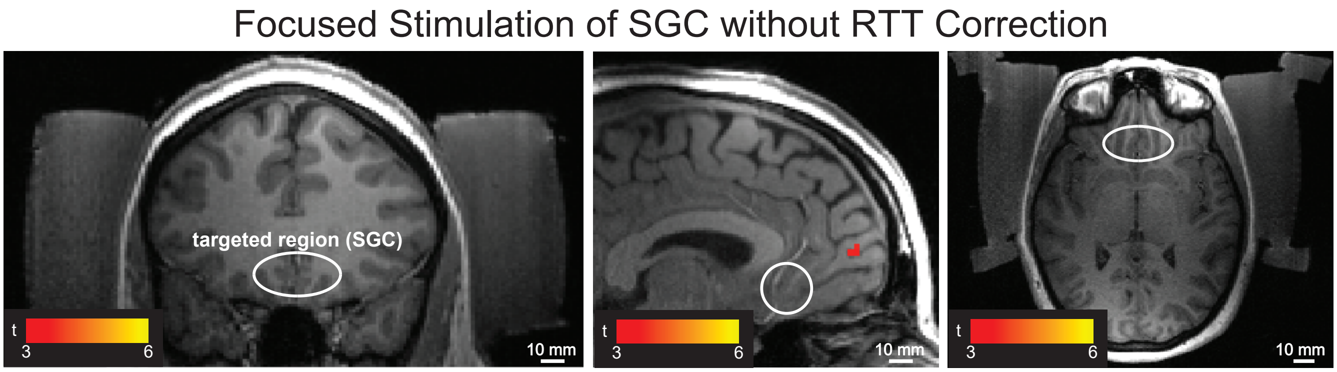

Supplementary Figure 11. RTT is critical for significant modulation of deep brain regions in humans. Same format as in Fig. 4 but without RTT applied. These data were obtained in Subject A.

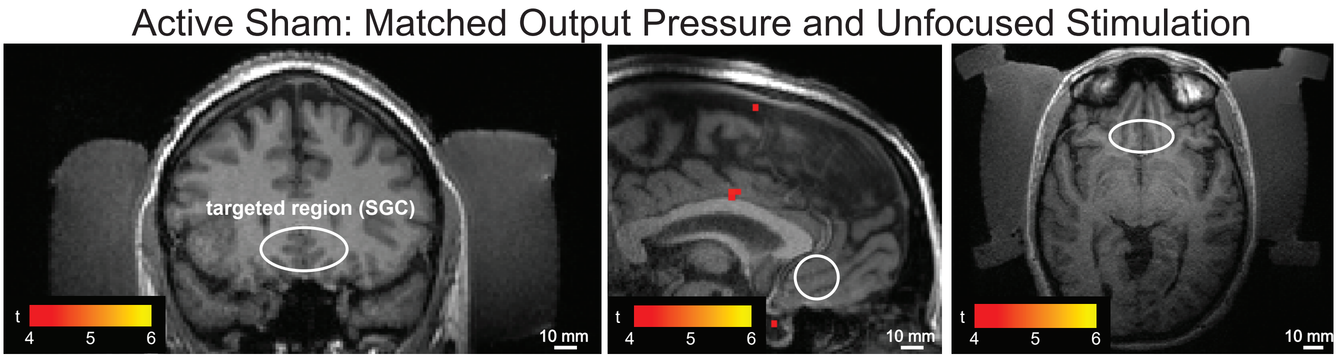

Supplementary Figure 12. Active sham control. To control for potential generic confounds that could be associated with ultrasound, we delivered into the brain a stimulus of the same levels and same timing but such that it was not focused (i.e., the transducers emitted a plane wave). Same format as in Fig. 4. There was no significant modulation ($p > 0.05$) of the target region in this case.

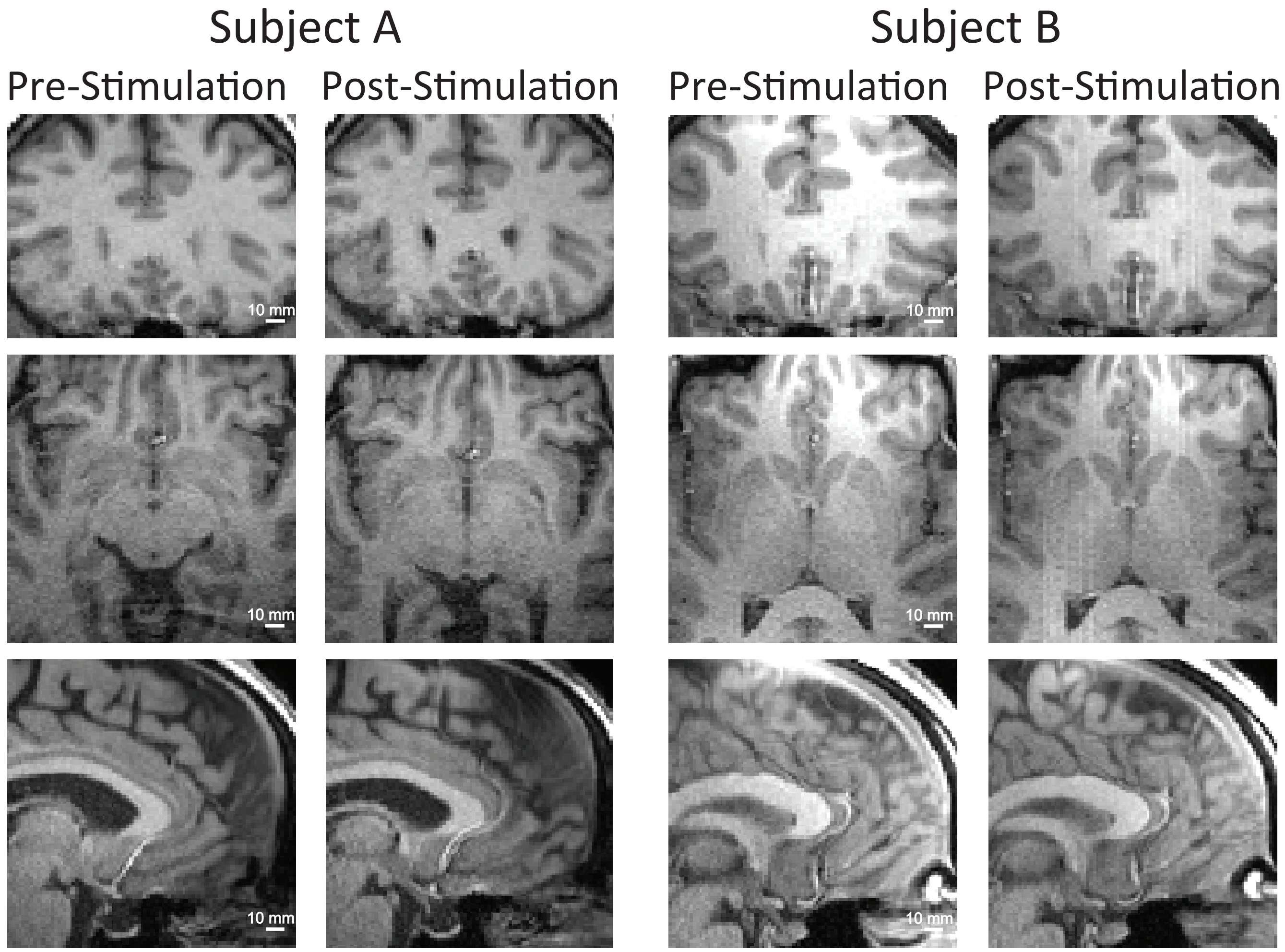

Supplementary Figure 13. T1w MRI before and after stimulation. T1-weighted MRI images taken before the stimulation and after the stimulation in each subject. There were no detectable signs of damage.

Adverse Effects Related To Treatment	Subject A	Subject B	Adverse Effects Related To Treatment	Subject A	Subject B
Headache	No	No	Skin rash or itching	No	No
Dry mouth	No	No	Tendency to develop bruises	No	No
Dizziness	No	No	Fever, increased temperature	No	No
Chest pain	No	No	Abnormal sweating	No	No
Palpitations	No	No	Hot flashes	No	No
Breathing problems	No	No	Convulsions or seizures	No	No
Circulation problems	No	No	Fatigue, loss of energy	No	No
Abdominal pain	No	No	Tremor	No	No
Nausea	No	No	Insomnia, sleeping problems	No	No
Vomiting	No	No	Back pain	No	No
Constipation	No	No	Muscle pain	No	No
Diarrhea	No	No	Joint pain	No	No
Reduced appetite	No	No	Agitation	No	No
Increased appetite	No	No	Irritability, nervousness	No	No
Difficulty urinating	No	No	Depressed mood	No	No
Sexual problems	No	No	Thoughts about suicide	No	No
Painful or irregular menstruation	No	No	Anxiety, fearfulness	No	No

Table S1. The stimulation was safe without adverse effects. Following the stimulation, patients were asked to complete a clinical questionnaire that assessed potential side effects. The options were 'No', 'Maybe', and 'Yes'.
