# Supplementary material for: Controlled noninvasive modulation of deep brain regions in humans
Source: Commun Eng. 2024 Jan 12;3:13. doi: 10.1038/s44172-023-00146-4 (PMC10956068; doi:10.1038/s44172-023-00146-4)
Supplement: Supplementary file 3 — Reporting Summary [file 44172_2023_146_MOESM3_ESM.pdf]

nature portfolio

Corresponding author(s): Tom Riis

Last updated by author(s): Nov 15, 2023

Reporting Summary

Nature Portfolio wishes to improve the reproducibility of the work that we publish. This form provides structure for consistency and transparency in reporting. For further information on Nature Portfolio policies, see our Editorial Policies and the Editorial Policy Checklist.

Statistics

For all statistical analyses, confirm that the following items are present in the figure legend, table legend, main text, or Methods section.

n/a	Confirmed	
☐	☒	The exact sample size (*n*) for each experimental group/condition, given as a discrete number and unit of measurement
☐	☒	A statement on whether measurements were taken from distinct samples or whether the same sample was measured repeatedly
☐	☒	The statistical test(s) used AND whether they are one- or two-sided<br>*Only common tests should be described solely by name; describe more complex techniques in the Methods section.*
☐	☒	A description of all covariates tested
☐	☒	A description of any assumptions or corrections, such as tests of normality and adjustment for multiple comparisons
☐	☒	A full description of the statistical parameters including central tendency (e.g. means) or other basic estimates (e.g. regression coefficient) AND variation (e.g. standard deviation) or associated estimates of uncertainty (e.g. confidence intervals)
☒	☐	For null hypothesis testing, the test statistic (e.g. *F*, *t*, *r*) with confidence intervals, effect sizes, degrees of freedom and *P* value noted<br>*Give P values as exact values whenever suitable.*
☒	☐	For Bayesian analysis, information on the choice of priors and Markov chain Monte Carlo settings
☒	☐	For hierarchical and complex designs, identification of the appropriate level for tests and full reporting of outcomes
☒	☐	Estimates of effect sizes (e.g. Cohen's *d*, Pearson's *r*), indicating how they were calculated

Our web collection on statistics for biologists contains articles on many of the points above.

Software and code

Policy information about availability of computer code

Data collection	All data collection was performed in MATLAB. We use open source code provided by manufacturere to collect data with oscilloscope (PicoScope 5244A). Code associated with data collection is available upon request.
Data analysis	All data analysis code was written in MATLAB. The analysis code makes use of the SPM12 open source software and matlab statistics toolbox. Code associated with the data analysis is available upon request.

For manuscripts utilizing custom algorithms or software that are central to the research but not yet described in published literature, software must be made available to editors and reviewers. We strongly encourage code deposition in a community repository (e.g. GitHub). See the Nature Portfolio guidelines for submitting code & software for further information.

Data

Policy information about availability of data

All manuscripts must include a data availability statement. This statement should provide the following information, where applicable:

- Accession codes, unique identifiers, or web links for publicly available datasets
- A description of any restrictions on data availability
- For clinical datasets or third party data, please ensure that the statement adheres to our policy

The data associated with the measurements are provided in the article. For raw data, contact the corresponding author.

Human research participants

Policy information about studies involving human research participants and Sex and Gender in Research.

Reporting on sex and gender	Data on all subject's sex was collected and report.
Population characteristics	Subject's age is reported in the text.
Recruitment	Human subjects that received stimulation were recruited from the Huntsman Mental Health Institute at the University of Utah by a clinician. Subjects where through transmit measurements were measured were recruited in person from the Biomedical Engineering Department at the University of Utah.
Ethics oversight	The University of Utah Institutional Review Board granted all human subject research as a non-significant risk. Protocol numbers are reported in the text.

Note that full information on the approval of the study protocol must also be provided in the manuscript.

Field-specific reporting

Please select the one below that is the best fit for your research. If you are not sure, read the appropriate sections before making your selection.

☒ Life sciences ☐ Behavioural & social sciences ☐ Ecological, evolutionary & environmental sciences

For a reference copy of the document with all sections, see nature.com/documents/nr-reporting-summary-flat.pdf

Life sciences study design

All studies must disclose on these points even when the disclosure is negative.

Sample size	Sample size for number of skulls was chosen to match or exceed previous studies of ultrasound skull correction methods (Leung et al 2021, Almquist et al. 2016, Miller et al. 2015).
Data exclusions	No data was excluded from the analysis.
Replication	We repeated the experiments in multiple independent subjects to ensure results were replicable.
Randomization	No relevant to study.
Blinding	Experimenters and persons performing the analysis were blinding to whether subjects received real or sham stimulation.

Reporting for specific materials, systems and methods

We require information from authors about some types of materials, experimental systems and methods used in many studies. Here, indicate whether each material, system or method listed is relevant to your study. If you are not sure if a list item applies to your research, read the appropriate section before selecting a response.

Materials & experimental systems

n/a	Involved in the study	
☒	☐	Antibodies
☒	☐	Eukaryotic cell lines
☒	☐	Palaeontology and archaeology
☒	☐	Animals and other organisms
☐	☒	Clinical data
☒	☐	Dual use research of concern

Methods

n/a	Involved in the study	
☒	☐	ChIP-seq
☒	☐	Flow cytometry
☐	☒	MRI-based neuroimaging

Clinical data

Policy information about clinical studies

All manuscripts should comply with the ICMJE guidelines for publication of clinical research and a completed CONSORT checklist must be included with all submissions.

Clinical trial registration	NCT05301036

Study protocol	Full trial can be accessed at the following link: https://clinicaltrials.gov/study/NCT05301036?term=Kubanek&rank=2
Data collection	Data will be collected at the University of Utah from July 2022 to July 2024
Outcomes	Our primary outcome measures are target engagment with MRI quantification of brain activation and mood effects quantified with Hamilton Depression Rating Scale, Positive and Negative Effect Schedule Extended (PANAS-X), and GAD-7.

Magnetic resonance imaging

Experimental design

Design type	Block Design
Design specifications	5 blocks of 1 minute stimulation interleaved with 1 minute of no stimulation. One 10 minute block per subject of both real stimulation and sham stimulation administered in double blind randomized fashion.
Behavioral performance measures	N.A.

Acquisition

Imaging type(s)	Functional
Field strength	3
Sequence & imaging parameters	Interleaved series, P-A phase encoding, TR 2.0 s, TE 33 ms, FA 80 degrees, FOV 207 mm, 52 slices, slice thickness 2.4 mm, bandwidth 2004 Hz/pixel, echo spacing 0.62 ms, 300 volumes per 10 minutes.
Area of acquisition	Whole Brain

Diffusion MRI ☐ Used ☒ Not used

Preprocessing

Preprocessing software	The processing consisted of four standard steps: i) co-registration of anterior to posterior and posterior to anterior field map to time-series (ANIMA) ii) echo-planar imaging (EPI) distortion correction (ANIMA) iii) realignment of time-series data (SPM12), and iv) application of Gaussian smoothing using a 8 mm kernel (SPM12).
Normalization	Data was not normalized as it was an individual subject analysis.
Normalization template	Data not normalized
Noise and artifact removal	Echo-planar imaging (EPI) distortion correction (ANIMA) and realignment of time-series data (SPM12)
Volume censoring	We preformed skull stripping in freesurfer to create a brain volume mask for all analyses.

Statistical modeling & inference

Model type and settings	Significance was determined using a false discovery rate correction with a p-value of <0.001.Minimum cluster size was set at 30 voxels. Standard general linear model regressed the stimulation factor (i.e., the blocks of 1-minute ON and 1-minute OFF stimulation) on the BOLD activity. The statistical difference between the ON and OFF outputs was assessed using a t-test.
Effect(s) tested	t-test between the ON and OFF conditions

Specify type of analysis: ☒ Whole brain ☐ ROI-based ☐ Both

Statistic type for inference (See Eklund et al. 2016)	Cluster wise with with minimum cluster size of 30 voxels.
Correction	False Discovery Rate corrected

Models & analysis

n/a	Involved in the study
☒	☐ Functional and/or effective connectivity
☒	☐ Graph analysis
☒	☐ Multivariate modeling or predictive analysis
